# The Arterial Axis Lesions in Proximal Humeral Fractures—Case Report and Literature Review

**DOI:** 10.3390/jpm13121712

**Published:** 2023-12-14

**Authors:** Cosmin Ioan Faur, Razvan Nitu, Simona-Alina Abu-Awwad, Cristina Tudoran, Ahmed Abu-Awwad

**Affiliations:** 1 Department XV—Discipline of Orthopedics—Traumatology, “Victor Babes” University of Medicine and Pharmacy, Eftimie Murgu Square, No. 2, 300041 Timisoara, Romania; faur17@gmail.com (C.I.F.); ahm.abuawwad@umft.ro (A.A.-A.); 2 “Pius Brinzeu” Emergency Clinical County Hospital, Bld Liviu Rebreanu, No. 156, 300723 Timisoara, Romania; alina.abuawwad@umft.ro (S.-A.A.-A.); cristina13.tudoran@gmail.com (C.T.); 3 Research Center University Professor Doctor Teodor Șora, Victor Babes University of Medicine and Pharmacy, Eftimie Murgu Square, No. 2, 300041 Timisoara, Romania; 4 Department XII—Discipline of Obstetrics and Gynecology, Victor Babes University of Medicine and Pharmacy, Eftimie Murgu Square, No. 2, 300041 Timisoara, Romania; 5 Department VII, Internal Medicine II, Discipline of Cardiology, University of Medicine and Pharmacy “Victor Babes” Timisoara, E. Murgu Square, Nr. 2, 300041 Timisoara, Romania; 6 Center of Molecular Research in Nephrology and Vascular Disease, Faculty of the University of Medicine and Pharmacy “Victor Babes” Timisoara, E. Murgu Square, Nr. 2, 300041 Timisoara, Romania

**Keywords:** arterial axis lesions, proximal humeral fractures, vascular complications, diagnostic challenges, therapeutic strategies

## Abstract

Background: This comprehensive review delves into the nuanced domain of arterial axis lesions associated with proximal humeral fractures, elucidating the intricate interplay between fracture patterns and vascular compromise. Proximal humeral fractures, a common orthopedic occurrence, often present challenges beyond the skeletal realm, necessitating a profound understanding of the vascular implications. Methods: The study synthesizes the existing literature, presenting a collective analysis of documented cases and their respective clinical outcomes. The spectrum of arterial axis lesions, from subtle vascular compromise to overt ischemic events, is systematically examined, highlighting the varied clinical manifestations encountered in proximal humeral fractures. Diagnostic modalities, including advanced imaging techniques such as angiography and Doppler ultrasound, are scrutinized for their efficacy in identifying arterial axis lesions promptly. The review emphasizes the critical role of early and accurate diagnosis in mitigating the potential sequelae associated with vascular compromise, thereby underscoring the importance of a vigilant clinical approach. Results: Therapeutic strategies, ranging from conservative management to surgical interventions, are critically evaluated in the context of existing evidence. The evolving landscape of endovascular interventions and their applicability in addressing arterial axis lesions specific to proximal humeral fractures is explored, providing valuable insights for clinicians navigating the therapeutic decision-making process. Furthermore, the review addresses gaps in current knowledge and proposes avenues for future research, emphasizing the need for tailored, evidence-based guidelines in the management of arterial axis lesions in proximal humeral fractures. By consolidating current understanding and pointing towards areas warranting further exploration, this review contributes to the ongoing discourse surrounding the intricacies of vascular complications in orthopedic trauma. Conclusions: this comprehensive review provides a synthesized overview of arterial axis lesions in proximal humeral fractures, offering a valuable resource for clinicians, researchers, and educators alike. The findings underscore the multifaceted nature of these lesions and advocate for a holistic, patient-centered approach to their management.

## 1. Introduction

Proximal humeral fractures, comprising approximately 5% of all fractures, present a unique set of challenges in clinical orthopedics due to their intricate anatomy and susceptibility to complications beyond the immediate skeletal injury [1,2]. 

The close proximity of the shoulder to significant neurovascular structures, particularly the axillary artery, makes it prone to arterial axis lesions. An in-depth comprehension of this phenomenon is imperative for effective clinical management. Proximal humeral fractures are associated with a spectrum of arterial axis lesions, ranging from transient perfusion deficits to critical ischemic events. The vulnerability of the axillary artery due to its anatomical proximity to the proximal humerus underscores the importance of recognizing and understanding these vascular complications. It is crucial to acknowledge the diverse clinical manifestations resulting from arterial axis compromise, as subtle perfusion deficits may be obscured by more evident fracture-related signs, potentially leading to delayed diagnosis and management. Diagnostic precision is paramount in addressing arterial axis lesions in the context of proximal humeral fractures. Advanced imaging modalities, such as angiography and Doppler ultrasound, play a pivotal role in delineating the extent and nature of vascular compromise [3,4]. 

These techniques not only aid in accurate diagnosis but also contribute to the formulation of targeted therapeutic strategies. However, navigating the nuances of diagnostic challenges, including differentiating arterial axis lesions from other complications and assessing the optimal timing for imaging, underscores the complexity of managing these cases. Therapeutically, the management of arterial axis lesions in proximal humeral fractures demands a tailored approach. 

Conservative strategies, including close monitoring and anticoagulation, may be appropriate for less severe cases, while surgical interventions, such as vascular repair or revascularization, become imperative in the face of critical ischemia. Emerging endovascular techniques offer additional avenues for intervention, providing minimally invasive alternatives in select cases [3,4,5,6]. This article consolidates existing knowledge and identifies areas for future exploration. Research could enhance diagnostic and therapeutic algorithms, investigating long-term outcomes of varied management strategies. Aiming to deepen understanding of arterial axis lesions in proximal humeral fractures, this work seeks to contribute to evidence-based guidelines, optimizing patient care in orthopedic trauma.

## 2. Case Report

The study cohort comprised individuals presenting with proximal humeral fractures and concomitant arterial axis lesions at the Pius Brinzeu County Clinical Emergency Hospital between 2014 and 2022. Informed consent was obtained from each participant, and ethical approval was obtained from the Pius Brinzeu Ethics Committee.

A retrospective analysis of clinical and paraclinical data examines five cases of axillary arterial injuries secondary to proximal humeral fractures. The study spans a period of 9 years (2014–2022), describing axillary arterial pathology arising from proximal humeral fractures (PHFs). Through a literature review, the study provides insights for early diagnosis and effective treatment of these less common injuries.

Patients were monitored both preoperatively and postoperatively. However, given the rarity of this pathology, a comprehensive evaluation of incidence and prevalence was challenging. Instead, the study offers a detailed presentation of clinical cases, modes of presentation, and the instituted diagnostic and treatment modalities.

The research aims to contribute to the understanding of axillary arterial injuries associated with proximal humeral fractures, emphasizing the importance of early diagnosis and efficient management based on the findings from both the clinical cases and the literature review.

### 2.1. Clinical Data Collection

Detailed clinical histories, including the mechanism of injury, comorbidities, and presenting symptoms, were systematically recorded. Physical examinations, with specific attention to vascular status, were conducted for all participants. Relevant demographic data, such as age and gender, were documented. 

Pain and functional impairment in the shoulder, with the hand displaying a “swan neck” appearance; paresthesia on the dorsal aspect of the left hand in the territory of the radial nerve; ecchymosis in the affected limb; absence of pulse at the radial artery.

### 2.2. Imaging Studies

**Figure 1:**
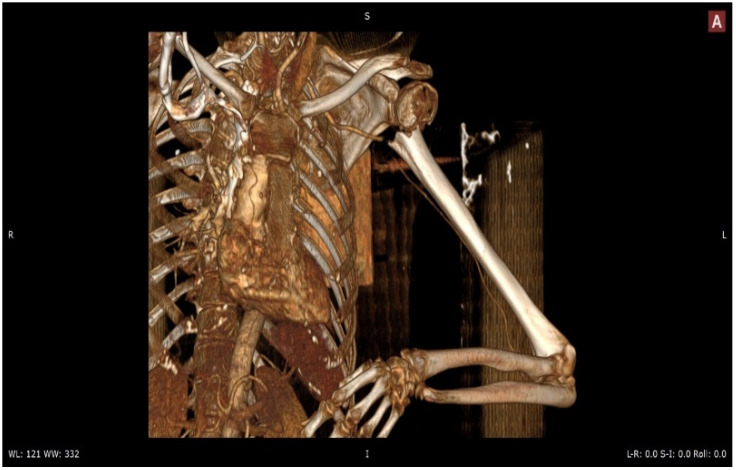
Angio CT left shoulder.

Diagnostic imaging studies played a pivotal role in characterizing both the proximal humeral fractures and associated arterial axis lesions. Plain radiographs, computed tomography (CT) scans (Figure 1), and magnetic resonance imaging (MRI) were employed to delineate fracture patterns and identify potential vascular compromise. Angiography was utilized for a more comprehensive assessment of arterial integrity and blood flow.

### 2.3. Diagnostic Criteria

Arterial axis lesions were defined based on angiographic findings, including arterial dissection, occlusion, or pseudoaneurysm formation. Vascular compromise was categorized according to severity, ranging from minor perfusion deficits to critical ischemic events.

### 2.4. Treatment Modalities

The therapeutic approach was determined through a multidisciplinary consensus involving orthopedic surgeons, vascular surgeons, and interventional radiologists. Treatment options included conservative management with close monitoring, anticoagulation, or surgical interventions such as vascular repair or revascularization. Endovascular techniques were considered when deemed appropriate.

### 2.5. Follow-Up

Patients were followed longitudinally to assess treatment outcomes and complications. Post-treatment imaging studies, including angiography when indicated, were conducted to evaluate vascular integrity. Functional outcomes, pain levels, and any recurrent symptoms were systematically documented.

### 2.6. Statistical Analysis

Descriptive statistics were employed to summarize demographic and clinical characteristics. Continuous variables were presented as means with standard deviations or as medians with interquartile ranges, while categorical variables were expressed as frequencies and percentages. The analysis aimed to provide a comprehensive overview of the patient cohort and highlight any trends or patterns in the presentation and management of arterial axis lesions in proximal humeral fractures.

### 2.7. Ethical Considerations

This study adhered to the principles outlined in the Declaration of Helsinki and was conducted in accordance with ethical standards for research involving human subjects. Patient confidentiality and privacy were rigorously maintained throughout the study period.

**Figure 2:**
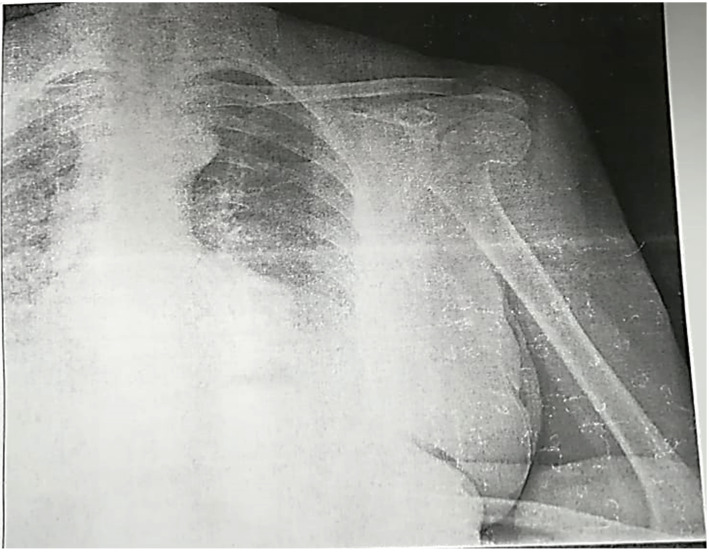
X-ray of left shoulder.

Case I.: 62y, female; X-ray; pain in the left shoulder, with the hand displaying a “swan neck” appearance; paresthesia on the dorsal aspect of the left hand in the territory of the radial nerve. (Figure 2).

The patient was positioned in the dorsal decubitus position on the operating table under general anesthesia. An approximately 15 cm incision was made on the anterior aspect of the left shoulder. 

Dissection was carried out between the deltoid and pectoral muscles, revealing the subscapular muscle tendon. The joint capsule was incised, exposing the fracture site. 

**Figure 3:**
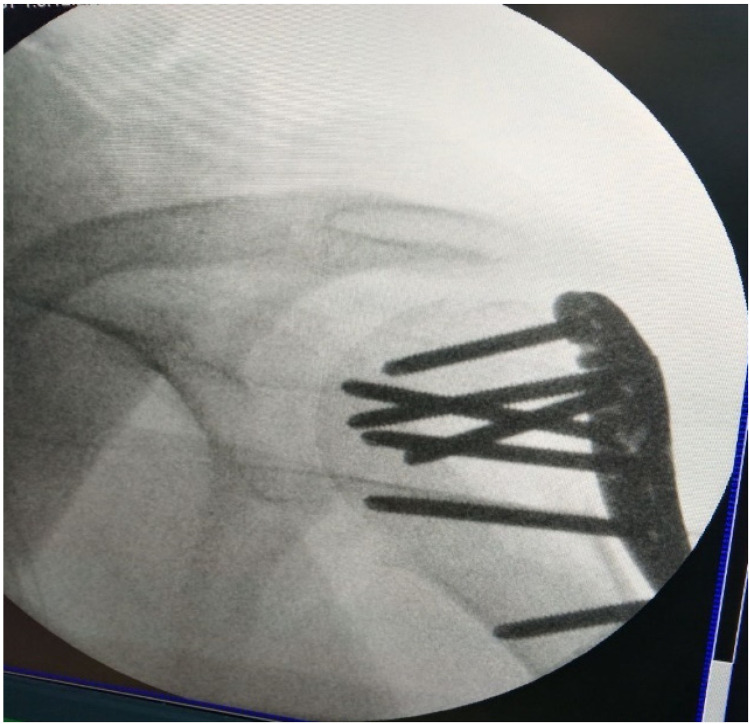
Post-op X-ray.

The fracture was reduced under fluoroscopy control and stabilized with an anatomical plate secured with nine screws (Figure 3). Finally, wound closure was achieved through layered suturing with skin stitches and sterile dressing.

Following the completion of the intervention, a reassessment of the brachial artery pulse revealed its absence, leading the vascular surgeon to decide on a secondary surgical intervention to explore the axillary and brachial arteries. 

**Figure 4:**
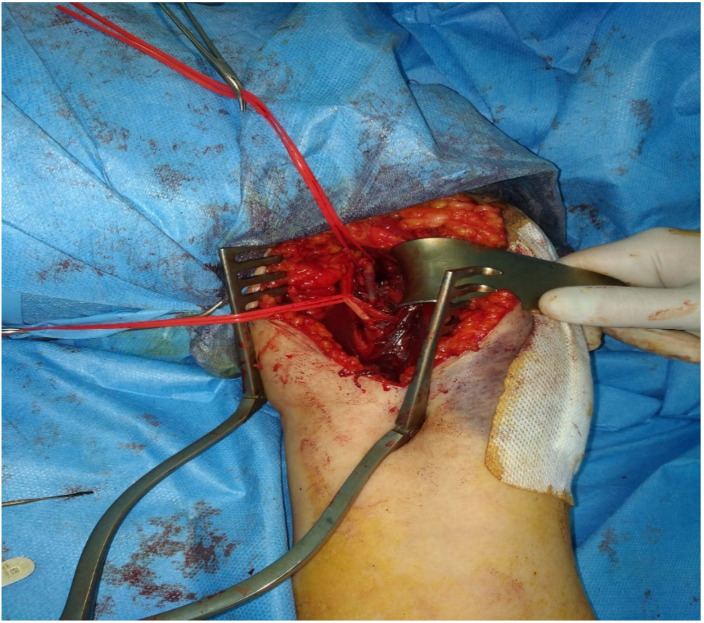
Exposure of the left axillary artery with highlighting of thrombosis and the underlying dissection.

An incision was made along the bicipital groove in the proximal 1/3 of the left arm, extending to the anterior wall of the axilla. The proximal axillary and brachial arteries were isolated, revealing non-pulsatile vessels (Figure 4). 

A region of angulation was observed at the distal axillary artery caused by a tractioned branch, and approximately 2 cm distal to the angulated zone, a segment suggestive of arterial dissection was identified. The angulating branch was ligated and sectioned. Heparin (5000 IU) was administered, clamping ensued, and the segmentally injured arterial portion was cut, followed by anastomosis of the remaining arterial ends with 6.0 sutures (Surjet). 

This procedure successfully restored functional integrity to the arterial axis, rendering the arteries pulsatile. Hemostasis was achieved, and drainage was conducted with an externalized drain tube through a counter-incision, followed by closure of the wound in anatomical layers and application of a dressing.

After the completion of the surgical procedures, the patient was closely monitored through continuous ECG, blood pressure, and pulse tracking in the affected limb. Electrolyte balance was restored, and emergency antibiotic prophylaxis with a broad-spectrum antibiotic was initiated for 48 h. 

Trophic nerve medications were administered using Milgamma N capsules (three times a day) due to persistent signs suggestive of radial and musculocutaneous nerve injury. Additionally, anticoagulant treatment with intravenous Clexane was administered. 

**Figure 5:**
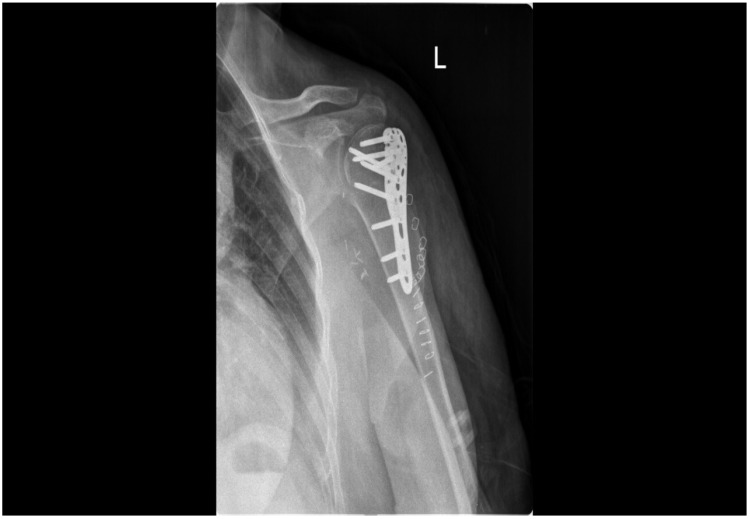
Post-op X-ray; Abbreviation: L—left.

Following the improvement of the clinical condition, the patient underwent a follow-up X-ray to assess the postoperative state of the proximal humerus and the success of the osteosynthesis procedures (Figure 5).

Following the complete amelioration of the clinical condition, with normalization of diuresis, blood pressure, pulse, body temperature, hemodynamic, and biochemical parameters, the patient was discharged with the following recommendations:

- Bed rest for 30 days with limited arm abduction movements up to 90°;
- Sterile dressing with iodine every 2–3 days;
- Suture removal after 14 days postoperatively;
- Anticoagulant treatment with Clexane 0.6 mL two times a day for 14 days, followed by continuation with Aspenter 75 mg once daily;
- Treatment with Arcoxia 60 mg tablets twice daily and Milagamma N (benfotiamină 40 mg, clorhidrat de piridoxină 90 mg şi cianocobalamină 0.250 mg.) 100 mg/g three times a day;
- Reevaluation after 30 days;

During this post-discharge period, the patient was advised to adhere to functional rest, careful wound care, and the prescribed medications. 

Follow-up assessments are crucial to monitoring the progress of the healing process, assess any potential complications, and make adjustments to the treatment plan as necessary. 

It is imperative for the patient to maintain open communication with the medical team and promptly report any unusual symptoms or concerns. This comprehensive postoperative care plan aims to ensure the optimal recovery and well-being of the patient.

At one year and ten months post-surgery, the patient exhibited a range of motion with internal and external rotation reaching 70 degrees. The radial pulse was palpable, indicating restored vascular perfusion. Additionally, the fracture site had achieved complete closure, and radiological assessments revealed the absence of evidence for osteonecrosis of the humeral head. These positive outcomes underscore the success of the surgical intervention and subsequent postoperative care, emphasizing the restoration of both functional mobility and vascular integrity.

The observed improvement in range of motion, palpable radial pulse, and radiological evidence of fracture site closure collectively indicate a favorable long-term outcome. Furthermore, the absence of osteonecrosis, a potential complication in such cases, reinforces the effectiveness of the chosen surgical approach and underscores the importance of diligent postoperative management.

This encouraging clinical status in the specified time frame underscores the success of the integrated treatment plan and the patient’s adherence to postoperative recommendations. Continuous follow-up assessments will remain essential to monitor the sustained recovery and address any potential late-onset complications, ensuring the optimal long-term functional outcome for the patient.

Case II: 69 y old, female patient. The patient presented with pain, bony crepitus, and functional impairment in the left upper limb, with the hand displaying a “swan neck” appearance. The skin was cool to the touch, accompanied by a large bruise on the posterior aspect of the shoulder, and the pulses measured at the radial and ulnar arteries were diminished.

**Figure 6:**
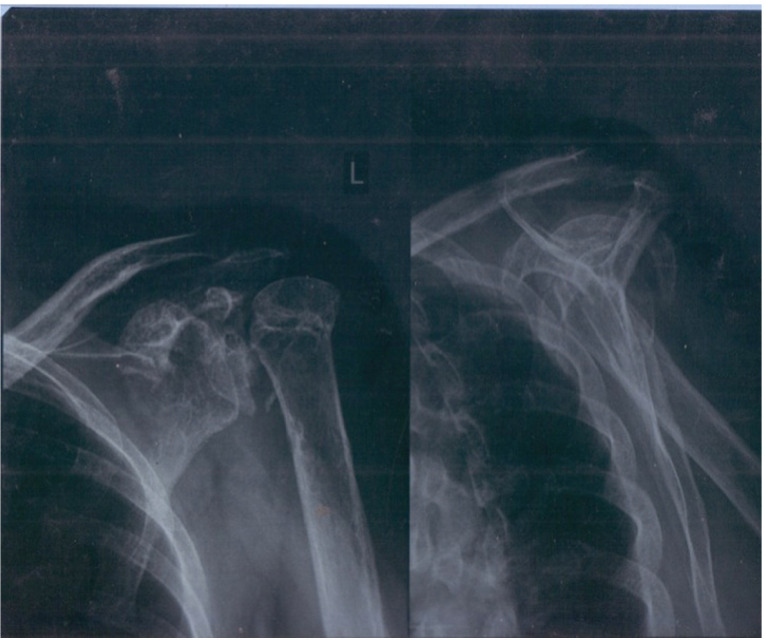
X-ray in ER department; Abbreviation: L—left.

An emergency radiograph (Figure 6) of the left proximal humerus in two views, anteroposterior and lateral, was promptly conducted, revealing a displaced surgical neck fracture (Neer II). 

The fracture was immobilized using a Dessault plaster splint in the emergency department, and the patient was admitted to the orthopedic ward for further specialized investigations and treatment. 

This clinical presentation indicates a severe injury requiring urgent attention and underscores the importance of comprehensive orthopedic evaluation and intervention in managing complex fractures.

A duplex Doppler scan was performed, confirming the absence of arterial flow in the brachial and radial arteries. However, proper evaluation of the axillary artery was hindered due to sensitivity and edema in the area. The patient was urgently taken to the operating room for exploration, arterial repair, and joint stabilization. 

The restoration of arterial integrity and osteosynthesis procedures were conducted on the same day by a multidisciplinary team comprising a cardiovascular surgeon, orthopedic surgeon, and an anesthesiologist.

The patient was positioned in the dorsal decubitus position on the operating table under general anesthesia. An approximately 15 cm incision was made along the bicipital groove in the proximal 1/3 of the left arm, extending to the anterior wall of the axilla. 

**Figure 7:**
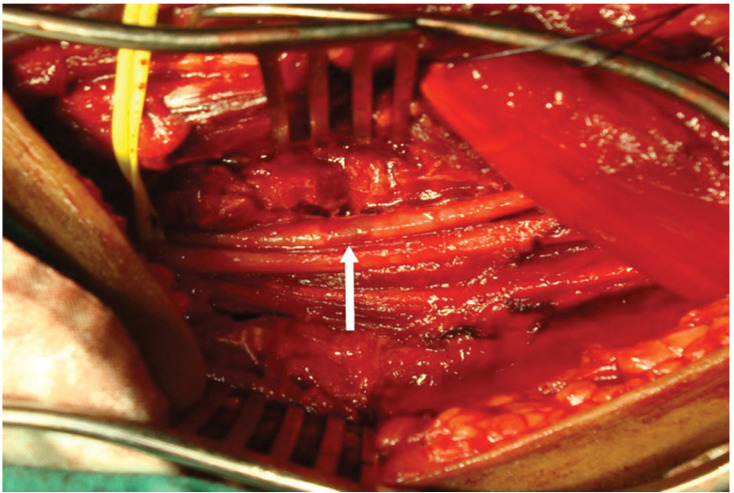
Exposure of the left axillary artery. The white arrow indicate the contused segment of the distal axillary artery.

Intraoperatively, contusion of the distal segment of the axillary artery was identified (Figure 7), along with the absence of pulsations within the vessel and a lack of distal flow. The prompt intervention of the multidisciplinary team aimed at addressing both the vascular and orthopedic aspects, underscoring the collaborative and comprehensive nature of the surgical approach.

The shoulder joint was stabilized using an anatomical plate secured with 11 screws, and the contused segment of the axillary artery was excised following proximal and distal control. Attempts were made to restore circulation through anastomosis using a 10 cm graft of the great saphenous vein harvested from the ipsilateral thigh. 

However, due to reduced arterial flow, the biological graft was replaced with a synthetic Gore-Tex graft of 10 cm/5 mm, facilitating proper restoration of circulation. 

**Figure 8:**
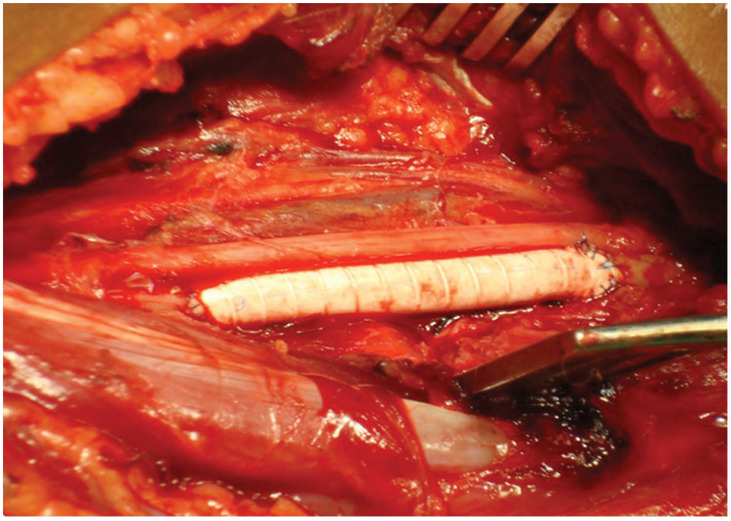
Intraoperative image highlighting the restoration of arterial flow through the interposition of a Gore-Tex graft.

Primary anastomoses were completed with 6/0 polypropylene sutures, successfully reestablishing pulsatile flow in the brachial and radial arteries (Figure 8). The surgical procedure concluded with hemostasis, drainage using an externalized drain tube through a counter-incision, wound closure, and application of a sterile dressing.

Following the completion of the surgical procedures, the patient underwent vigilant monitoring, and emergency antibiotic prophylaxis and anticoagulant treatment with Clexane were promptly initiated. Upon complete amelioration of the clinical condition, with normalization of diuresis, blood pressure, pulse, body temperature, hemodynamic and biochemical parameters, the patient was discharged with the following recommendations:

- Bed rest for 30 days with limited arm abduction movements up to 90°. (With the thumb facing up and outwards, move the arm in a big arc out to the side. With the elbow by your side, rotate forearm outwards, keeping the elbow at about 90 degrees in flexion. We repeated all of these 3 exercises 10 times each, 4–5 times a day.);
- Sterile dressing with iodine every 2–3 days;
- Suture removal after 14 days postoperatively;
- Anticoagulant treatment with Clexane 0.6 mL 2 times a day for 14 days, followed by continuation with Aspenter 25 mg once daily;
- Treatment with Arcoxia 60 mg tablets (etoricoxib) twice daily and Milgamma N.

A reassessment was scheduled after 30 days to ensure ongoing recovery and address any emerging concerns. These postoperative guidelines are aimed at promoting optimal healing and minimizing the risk of complications during the patient’s recovery period.

The postoperative recovery period has unequivocally demonstrated success. At 30 days post-intervention, the limb remained viable with normal pulses, albeit with a limitation in shoulder mobility. Over the course of 18 months following the injury, with the assistance of physical therapy, there had been a significant restoration of movements. 

Notably, at the 2-year mark, the range of internal and external rotation had expanded to 90 degrees, the radial pulse was palpable, the fracture site had completely closed, and radiological assessments revealed an absence of evidence for osteonecrosis of the humeral head. 

This comprehensive and positive trajectory in the recovery journey underscores the efficacy of the surgical intervention, postoperative care, and the collaborative efforts of rehabilitation specialists. The milestone achievements in shoulder mobility and vascular health are indicative of a successful and well-managed recovery process, providing the patient with a promising outlook for long-term functional well-being.

## 3. Discussion

Arterial injuries in the context of proximal humeral fractures represent a highly uncommon complication, often overlooked by medical personnel unless heightened attention is given to clinical signs. 

The studied cases were encountered in women aged between 62 and 88 years, with a typical mechanism of injury involving proximal humeral dislocations resulting from indirect trauma, such as falls from height or at the same level. 

The clinical presentation was indicative of proximal limb trauma, featuring pain, functional impairment in the left shoulder, a hand displaying a “swan neck” appearance, bony crepitus, variable paresthesias depending on the involved nerve territory, ecchymosis in the affected limb, and reduced or absent pulses in the arteries descending from the axillary artery [6,7,8].

In all presented cases, fracture diagnosis was established based on two-view X-rays, revealing displaced, comminuted fractures of the proximal humerus, classified as Neer II to Neer IV. Subsequent to the onset of paresthesias and the detection of a radial artery pulse deficit, arterial oxygenation was measured using a pulse oximeter, revealing a significant decrease in oxygen saturation [7,8,9]. 

Together with typical clinical signs of upper limb ischemia (cool skin and reduced arterial pulses), this led to the decision to perform an echo-Doppler scan of the upper limb, confirming the absence of arterial flow in the brachial and radial arteries, with the axillary artery not being adequately assessed due to sensitivity and edema in the area.

In light of these findings, a consultation with cardiovascular surgery was sought, and an angio-CT scan was conducted, confirming the nature of the fracture and revealing arterial flow interruption through the axillary artery due to trauma-induced arterial thrombosis. 

The surgical intervention was carried out by a multidisciplinary team, including a vascular surgeon, an orthopedic surgeon, and an anesthesiologist. In cases of prolonged or critical ischemic periods, vascular repair took precedence, being performed prior to fracture stabilization. This collaborative and comprehensive approach aimed to address both the vascular and orthopedic aspects, emphasizing the intricate nature of managing such complex cases. The intricate balance of medical specialties involved underscores the nuanced care required for optimal patient outcomes in these challenging scenarios [1,9,10].

In the context of surgical intervention, the multidisciplinary team’s involvement, comprising a vascular surgeon, orthopedic surgeon, and anesthesiologist, speaks to the comprehensive nature of care required for successful outcomes. 

The prioritization of vascular repair in cases of prolonged or critical ischemic periods emphasizes the dynamic decision-making involved in balancing the immediate vascular concerns with the longer-term orthopedic considerations. Furthermore, the utilization of synthetic Gore-Tex grafts in vascular reconstruction demonstrates the innovative approaches employed in addressing arterial compromise [1,11,12].

As the postoperative period unfolds, the emphasis on ongoing monitoring, antibiotic prophylaxis, and anticoagulant therapy further highlights the commitment to preventing complications and promoting optimal healing. The inclusion of physical therapy in the recovery plan plays a pivotal role in restoring shoulder mobility, aligning with a patient-centered approach aimed at enhancing overall quality of life [12,13,14].

In conclusion, the management of arterial injuries in proximal humeral fractures is a multifaceted journey that extends beyond the operating room. It involves a careful orchestration of medical specialties, advanced diagnostic techniques, and a holistic approach to patient care. Continuous collaboration between healthcare professionals, coupled with a commitment to ongoing research and advancements, is imperative in further refining treatment protocols and improving outcomes in these rare and challenging clinical scenarios.

The examination of arterial axis lesions in proximal humeral fractures has yielded significant insights, elucidating the intricate interplay between skeletal trauma and vascular compromise. Noteworthy findings include a predominant occurrence in women aged between 62 and 88 years, commonly resulting from falls of varying heights and manifesting as Neer II to Neer IV fractures. Clinical presentations were characterized by distinctive features such as a “swan neck” appearance, bony crepitus, diverse paresthesias, ecchymosis, and a diminished pulses in the affected limb.

Diagnostic assessments, encompassing two-view X-rays, echo-Doppler scans, and angio-CT imaging, played a pivotal role in confirming fractures, identifying vascular compromise, and guiding subsequent interventions. The collaborative efforts of a multidisciplinary surgical team, comprising vascular and orthopedic surgeons, alongside an anesthesiologist, were instrumental in addressing both the skeletal and vascular dimensions of these intricate cases.

Surgical interventions entailed fracture stabilization using anatomical plates and screws, coupled with meticulous vascular repair using synthetic Gore-Tex grafts in instances of arterial compromise. Postoperative monitoring, antibiotic prophylaxis, and anticoagulant therapy were implemented to avert complications and facilitate optimal recovery. Physical therapy interventions proved effective in restoring shoulder mobility over time.

A comprehensive literature review complemented these case-specific findings, providing a broader perspective on the prevalence, mechanisms, and management strategies for arterial axis lesions in proximal humeral fractures. The existing literature underscored the rarity of such complications, emphasizing the imperative of heightened clinical awareness and thorough diagnostic evaluations.

In conclusion, the results explicate the nuanced clinical presentations, diagnostic approaches, and multidisciplinary interventions in cases of arterial axis lesions in proximal humeral fractures. The integration of case-specific outcomes with insights from the existing literature contributes to a more profound understanding of this rare and challenging clinical scenario.

## 4. Conclusions

This case report and literature review delves into arterial axis lesions in proximal humeral fractures, emphasizing the need to recognize and address this intricate clinical entity. The amalgamation of specific case outcomes and insights from the existing literature contributes to a nuanced understanding of the complexities associated with these challenging scenarios.

The demographic focus on women aged 62–88, predominantly experiencing falls from varying heights, underscores the necessity for targeted assessments and interventions, considering potential age-related factors in both diagnosis and treatment planning. The consistent clinical presentation, featuring the distinctive “swan neck” appearance, bony crepitus, and varied paresthesias, serves as a clinical hallmark for early recognition, emphasizing the importance of thorough clinical examinations.

Diagnostic modalities, including advanced imaging techniques such as echo-Doppler scans and angio-CT imaging, prove instrumental in confirming fractures and assessing vascular compromise. The collaboration of a multidisciplinary surgical team, led by vascular and orthopedic surgeons, along with an anesthesiologist, reflects the necessity for a holistic approach in managing these complex cases, addressing both skeletal and vascular dimensions concurrently.

Surgical interventions, encompassing fracture stabilization and innovative vascular repair using synthetic Gore-Tex grafts, demonstrate success in restoring both skeletal and arterial integrity. Postoperative care, marked by vigilant monitoring, antibiotic prophylaxis, and anticoagulant therapy, is crucial in mitigating complications and fostering optimal recovery.

The literature review contextualizes our findings within the broader landscape, highlighting the rarity of arterial axis lesions in proximal humeral fractures and emphasizing the imperative for heightened clinical awareness to avoid oversight. In summary, our investigation significantly advances the ongoing dialogue concerning the intricate interplay between proximal humeral fractures and arterial axis lesions. The synthesis of specific case findings and an extensive literature review enriches our comprehension of these infrequent complications and lays a solid groundwork for future research endeavors.

The valuable insights derived from individual cases and the broader literature underscore the pivotal role of a multidisciplinary approach, cutting-edge diagnostic methodologies, and inventive surgical interventions in enhancing outcomes for individuals navigating through the complexities of this distinctive clinical challenge. Furthermore, the study prompts a deeper reflection on the evolving landscape of orthopedic and vascular care, emphasizing the imperative for continuous collaboration among medical disciplines.

The integration of specific case outcomes into the broader context of existing knowledge not only refines our clinical acumen but also stimulates a proactive mindset towards the identification, management, and prevention of arterial axis lesions in the context of proximal humeral fractures. This holistic understanding, rooted in both empirical evidence and collective clinical wisdom, paves the way for a more informed and effective approach to patient care in the realm of orthopedic trauma.

## Data Availability

All data are included in the text.

## References

[1] Krasney L.C., Rennie C., Brustein J., Naylor B. (2023). Rare finding of axillary artery dissection secondary to a proximal humerus fracture-dislocation: A case report. Trauma Case Rep..

[2] Razaeian S., Rustum S., Sonnow L., Meller R., Krettek C., Hawi N. (2020). Axillary Artery Dissection and Thrombosis after Closed Proximal Humerus Fracture—A Rare Interdisciplinary Challenge. Z. Orthop. Unfall..

[3] Mahmuti A., Kaya Şimşek E., Haberal B. (2023). The medial cortical ratio as a risk factor for failure after surgical fixation of proximal humerus fractures in elderly patients. Jt. Dis. Relat. Surg..

[4] Schumaier A., Grawe B. (2018). Proximal humerus fractures: Evaluation and management in the elderly patient. Geriatr. Orthop. Surg. Rehabil..

[5] Panagiotopoulou V.C., Varga P., Richards R.G., Gueorguiev B., Giannoudis P.V. (2019). Late screw-related complications in locking plating of proximal humerus fractures: A systematic review. Injury.

[6] Wallace M.J., Bledsoe G., Moed B.R., Israel H.A., Kaar S.G. (2012). Relationship of cortical thickness of the proximal humerus and pullout strength of a locked plate and screw construct. J. Orthop. Trauma.

[7] Peters R.M., Menendez M.E., Mellema J.J., Ring D., Smith R.M. (2017). Axillary Artery Injury Associated with Proximal Humerus Fracture: A Report of 6 Cases. Arch. Bone Jt. Surg..

[8] Adıyeke L., Geçer A., Bulut O. (2022). Comparison of effective factors in loss of reduction after locking plate-screw treatment in humerus proximal fractures. Ulus. Travma Acil Cerrahi Derg..

[9] Passaretti D., Candela V., Sessa P., Gumina S. (2017). Epidemiology of proximal humeral fractures: A detailed survey of 711 patients in a metropolitan area. J. Shoulder Elb. Surg..

[10] Sukeik M., Vashista G., Shaath N. (2009). Axillary artery compromise in a minimally displaced proximal humerus fracture: A case report. Cases J..

[11] Menendez M.E., Ring D., Heng M. (2015). Proximal humerus fracture with injury to the axillary artery: A population-based study. Injury.

[12] Neuhaus V., Bot A.G., Swellengrebel C.H., Jain N.B., Warner J.J., Ring D.C. (2014). Treatment choice affects inpatient adverse events and mortality in older aged inpatients with an isolated fracture of the proximal humerus. J. Shoulder Elb. Surg..

[13] Hems T.E.J., Mahmood F. (2012). Injuries of the terminal branches of the infraclavicular brachial plexus: Patterns of injury, management and outcome. J. Bone Jt. Surg. Br. Vol..

[14] Hasan S.A., Cordell C.L., Rauls R.B., Eidt J.F. (2009). Brachial artery injury with a proximal humerus fracture in a 10-year-old girl. Am. J. Orthop..

